# Effects of Resistance Exercise on Slow-Twitch Soleus Muscle of Infarcted Rats

**DOI:** 10.3390/antiox12020291

**Published:** 2023-01-27

**Authors:** Lidiane Moreira Souza, Mariana Janini Gomes, Bruna Brasil Brandao, Luana Urbano Pagan, Mariana Gatto, Felipe Cesar Damatto, Eder Anderson Rodrigues, Thierres Hernani Dias Pontes, Patricia Aparecida Borim, Ana Angelica Henrique Fernandes, Gilson Masahiro Murata, Leonardo Antonio Mamede Zornoff, Paula Schmidt Azevedo, Katashi Okoshi, Marina Politi Okoshi

**Affiliations:** 1 Department of Internal Medicine, Botucatu Medical School, Sao Paulo State University, UNESP, Botucatu 18618-687, SP, Brazil; 2 Department of Kinesiology and Sport Management, Texas A&M University, College Station, TX 77845, USA; 3 Section of Integrative Physiology and Metabolism, Joslin Diabetes Center, Harvard Medical School, Boston, MA 02215, USA; 4 Department of Chemistry and Biochemistry, Institute of Biosciences, Sao Paulo State University, UNESP, Botucatu 18618-970, SP, Brazil; 5 Laboratory of Medical Investigation (LIM-29), Clinic Medical Department, University of Sao Paulo Medical School, Sao Paulo 01246-903, SP, Brazil

**Keywords:** heart failure, myocardial infarction, physical training, rat, left ventricular function, echocardiogram

## Abstract

Although current guidelines recommend resistance exercise in combination with aerobic training to increase muscle strength and prevent skeletal muscle loss during cardiac remodeling, its effects are not clear. In this study, we evaluated the effects of resistance training on cardiac remodeling and the soleus muscle in long-term myocardial infarction (MI) rats. Methods: Three months after MI induction, male Wistar rats were assigned to Sham (*n* = 14), MI (*n* = 9), and resistance exercised MI (R-MI, *n* = 13) groups. The rats trained three times a week for 12 weeks on a climbing ladder. An echocardiogram was performed before and after training. Protein expression of the insulin-like growth factor (IGF)-1/protein kinase B (Akt)/rapamycin target complex (mTOR) pathway was analyzed by Western blot. Results: Mortality rate was higher in MI than Sham; in the R-MI group, mortality rate was between that in MI and Sham and did not differ significantly from either group. Exercise increased maximal load capacity without changing cardiac structure and left ventricular function in infarcted rats. Infarction size did not differ between infarcted groups. Catalase activity was lower in MI than Sham and glutathione peroxidase lower in MI than Sham and R-MI. Protein expression of p70S6K was lower in MI than Sham and p-FoxO3 was lower in MI than Sham and R-MI. Energy metabolism did not differ between groups, except for higher phosphofrutokinase activity in R-MI than MI. Conclusion: Resistance exercise is safe and increases muscle strength regardless structural and functional cardiac changes in myocardial-infarcted rats. This exercise modality attenuates soleus glycolytic metabolism changes and improves the expression of proteins required for protein turnover and antioxidant response.

## 1. Introduction

Cardiovascular disease is an important worldwide cause of morbidity and mortality [1]. Myocardial infarction (MI) is a highly prevalent cardiovascular disease and a major contributor in heart failure development [2]. Ischemia-induced myocardial injury leads to a chronic process known as post-MI cardiac remodeling, which is defined as the molecular, cellular, and interstitial changes that clinically manifest as alterations in heart shape, size, and function [3,4]. Despite improvements in medical therapy, the final outcome of pathological cardiac remodeling is heart failure.

In addition to affecting the heart, MI also induces changes in organs such as the liver and kidneys, and skeletal muscles. Alterations in skeletal muscles mainly occur in advanced heart failure and contribute to a reduced functional capacity and ability to perform physical activities [5,6,7]. Skeletal muscle abnormalities including widespread myopathy phenotype with atrophy, decreased insulin-like growth factor-1 (IGF-1) signaling, increased oxidative stress, and impaired functionality have been observed in experimental and clinical studies [8,9,10,11,12,13,14,15].

Physical exercise has displayed beneficial effects in experimental studies [16,17,18,19] and has been recommended as an adjuvant therapy for stable post-infarction or heart failure patients [20,21,22,23]. Exercise improves quality of life and reduces hospitalization [22,24]. Aerobic training was the preferred exercise as there is concern that increased intravascular pressure during weightlifting could increase intraventricular pressure and impair left ventricular (LV) dilation during cardiac remodeling [25,26,27]. However, considering that resistance training improves physical capacity, maximum oxygen consumption, endurance, muscle strength, and quality of life [26,28,29,30,31,32], it has started to be considered for patients with heart failure or post-myocardial infarction [33].

Current guidelines recommend resistance exercise in combination with aerobic training to improve functional capacity, increase muscle strength, and prevent or reverse skeletal muscle loss [22,34]. However, few studies have evaluated the effects of resistance exercise on skeletal muscles during cardiac remodeling [35,36].

In a previous study, we analyzed the effects of aerobic and resistance exercise on the gastrocnemius of infarcted rats [37]. Despite extensive evaluation of morphometry, oxidative stress, protein oxidation, and satellite cell activation, in addition to the expression of proteins of proteasome, ubiquitin, and Pax7, we found that resistance exercise only improved superoxide dismutase activity and attenuated oxidative stress. The gastrocnemius is predominantly composed of glycolytic fast fibers [37]. We have not identified studies evaluating the soleus muscle in a similar experimental model. The soleus is mainly composed of oxidative fibers [13]. Therefore, the soleus strongly relies on oxidative phosphorylation for proper function, thus being prone to present changes such as increased oxidative stress, protein degradation, apoptosis, necrosis, atrophy, and metabolic disturbance during MI or heart failure [13,38]. In this study, we evaluated the effects of resistance training on cardiac remodeling and the soleus muscle in long-term infarcted rats. Our analyzes focused on oxidative stress, cell metabolism, and the cell signaling pathways related to muscle atrophy.

## 2. Materials and Methods

### 2.1. Experimental Groups

Male Wistar rats weighing between 200–250 g were purchased from the Central Animal Center of Botucatu Medical School, UNESP. The animals were kept in collective cages with three animals each in a temperature-controlled room with a 12-h light–dark cycle. The experiments and procedures were approved by the Animal Experimentation Ethics Committee of Botucatu Medical School, UNESP, SP, Brazil, in accordance with the rules of the Brazilian College of Animal Experimentation.

MI was induced by ligating the left anterior descending coronary artery as previously described [17]. Three months later, rats underwent echocardiogram to ensure infarcted area homogeneity between groups and were assigned to three groups: Sham operated (*n* = 14), MI (*n* = 26), and resistance exercised MI (R-MI, *n* = 21). Physical training was maintained for 3 months. At the end of the experimental period, rats were subjected to transthoracic echocardiogram, exercise testing, and euthanized the next day. All surviving rats were included in the anatomical analyzes. One rat from Sham and one from R-MI group had a poor echocardiographic window and were excluded from this evaluation. Six to eight rat muscle samples were randomly chosen for biochemical and molecular analyzes.

### 2.2. Maximum Carrying Load Test

Maximum carrying load capacity was assessed on a vertical ladder (1.00 m height, 0.20 m width, 0.5-cm grid, and 80° inclination). Adaptive training consisted of rats performing three climbs from different points on the ladder: near the top, in the middle, and the bottom; these were repeated over three consecutive days [39]. Maximum carrying load was then evaluated for each rat by performing a maximum of nine ladder climbs with progressively heavier loads. On the first attempt, rats climbed the ladder carrying a load equivalent to 75% of their body weight. After completing each climb, the load was progressively increased by 15% of body weight until the rats could not climb the entire ladder. The heaviest load successfully carried the entire height of the ladder was considered the maximum carrying load. Failure was determined when the rat could not progress up the ladder after three successive stimuli to the tail [40]. Maximum carrying load test was performed before the training protocol, 45 days after training for load adjustment (Supplementary data, Table S1), and at the end of the experiment.

### 2.3. Resistance Exercise Training

Rats were subjected to the training protocol three non-consecutive days per week for 12 weeks. In the first week, the rats performed three climbs with gradually increasing loads: no load on the first day, 15% of rat body weight on the second day, and 30% of rat body weight on the third day. From the second week on, the protocol consisted of four climbs. The climbs consisted of each rat carrying progressive loads of 50%, 75%, 90%, and 100% of its maximal carrying load capacity with a 2-min rest between climbs in the housing chamber at the top of the ladder [37,41]. Rats were re-evaluated after 45 days to adjust the training load.

### 2.4. Echocardiographic Evaluation

After anesthesia by intramuscular injection with a mixture of ketamine (50 mg/kg) and xylazine (1 mg/kg), an echocardiogram was performed using an apparatus (Vivid S6, General Electric Medical Systems, Tirat Carmel, Israel) equipped with a 5–11.5 MHz multifrequency probe, as previously described [42,43,44,45]. This was performed both before and after physical training.

### 2.5. Collection of Skeletal Muscle and Other Tissues

Rats were anesthetized with intraperitoneal sodium thiopental (50 mg/kg) and euthanized. After blood collection, hearts were removed by thoracotomy. Atria and ventricles were dissected and weighed. Soleus muscles from the right and left hind limbs were dissected, weighed, frozen in liquid nitrogen, and stored at −80 °C.

### 2.6. Infarct Size

Samples from LV were fixed in a 10% buffered formalin solution for 24 h, then washed in water and transferred to a solution with ethanol, according to a previously described method [46]. To calculate infarction size, LV was cut at a distance of 5 to 6 mm from the apex, as left midventricular slices present a close linear relation with the sum of the measurements from all heart [46]. LV slices were stained with picrosirius red and examined under a compound microscope (Leica DM LS; Nussloch, Germany) coupled to a computerized imaging analysis system (Media Cybernetics, Silver Spring, MD, USA) [47]. Infarction size was calculated by dividing the sum of endocardial and epicardial infarcted ventricular lengths by the sum of the total (infarcted and viable myocardium) endocardial and epicardial ventricular circumferences [46]. Values were expressed as percentage of the total LV area. Only rats with MI > 30% of total LV area at histological evaluation were included in this study.

### 2.7. Skeletal Muscle Morphology

Serial transverse sections of the soleus muscles were cut at a 10-µm thickness in a cryostat cooled to −20 °C. The general morphology was evaluated in sections stained with hematoxylin and eosin. At least 150 cross-sectional fiber areas were measured from each soleus muscle [47].

### 2.8. Antioxidant Enzyme Activity and Lipid Hydroperoxide Concentration

Soleus samples (∼100 mg) were homogenized in 2 mL of cold 0.1 M phosphate buffer, pH 7.0. Tissue homogenates were prepared in a motor-driven Teflonglass Potter-Elvehjem, tissue homogenizer. The homogenate was centrifuged at 10,000× g for 15 min at 4 °C, and the supernatant was assayed for total protein, lipid hydroperoxide, and glutathione peroxidase (GSH-Px, E.C.1.11.1.9), catalase (E.C.1.11.1.6.), and superoxide dismutase (SOD, E.C.1.15.1.1.) activities by spectrophotometry [13]. Enzyme activities were analyzed at 25 °C using a microplate reader (𝜇Quant-MQX 200) with Kcjunior software for computer system control (Bio-Tec Instruments, Winooski, VT, USA). Spectrophotometric determinations were performed in a Pharmacia Biotech spectrophotometer with a temperature-controlled cuvette chamber (UV/visible Ultrospec 5000 with Swift II applications software for computer system control, Cambridge, UK). All reagents were purchased from Sigma Aldrich (St. Louis, MO, USA) [48,49].

### 2.9. Assessment of Energy Metabolism

Soleus samples (~20 mg) were used to determine the maximum activity of enzymes that participate in glucose metabolism: phosphofructokinase (PFK), pyruvate kinase (PK), lactate dehydrogenase (LDH), citrate synthase (CS), and carnitine palmitoyltransferase-1 (CPT-1), as previously described [50,51,52,53]. Enzyme activities were assessed in triplicate and measurements performed every 10 s over a 3 min period on Spectramax M5 spectrophotometer (Molecular Devices, Sunnyvale, CA, USA). Results were expressed on a protein basis as determined by the BCA protein assay kit (Thermo Fisher Scientific, Waltham, MA, USA).

### 2.10. Protein Expression

Western blotting was performed as previously described [46,54]. The primary antibodies used in this study were rabbit anti- IGF-1R (#9750s), AKT (#9272), phospho-AKTSer473 (#9271), mTOR (#2972), phospho-mTORSer2448 (#2971), p70 S6 kinase (#9202), phospho-p70Thr389 (#9205), FoxO3a (#2497), phospho-FoxO3aSer294 (#5538; Cell Signaling Technology, Danvers, MA, USA), and anti-IGF1 (H-70 sc-9013; Santa Cruz Biotechnology, Santa Cruz, CA, USA). Protein levels were normalized to GAPDH (6C5 sc-32233, Santa Cruz Biotechnology, Dallas, TX, USA). Soleus samples (~50 mg) were homogenized in 50 mM Tris-HCl, 1 mM EDTA and protease inhibitor (Sigma Ref. S8820-2TAB, Burlington, MA, USA), pH 7.4, using zirconium beads (0.5 mm) for 5 min at 4 °C in a Bullet Blender^®^ homogenizer (Next Advance, Inc., Troy, NY, USA). The lysate was centrifuged at 12,000 rpm for 10 min at 4 °C and supernatant protein content was quantified by the Bradford assay. Samples were separated on a polyacrylamide gel and transferred to a nitrocellulose membrane. After 1 h blockade, membrane was incubated with the primary antibodies (overnight at 4 °C), washed with TBS and Tween 20, and incubated with secondary peroxidase-conjugated antibodies for 90 min at room temperature. Immobilon^®^ Classico Western HRP Substrate (Merck Millipore, Ref. WBLUC0500, Burlington, MA, USA) and an Image Quant LAS 4000 image analyzer (GE Healthcare Life Sciences, Chicago, IL, USA) were used to detect bound antibodies, which were quantified by densitometry using Gel Pro 3.1.

### 2.11. Statistical Analysis

Data normality was evaluated by the Shapiro–Wilk test. Comparisons between groups were performed by one-way analysis of variance (ANOVA) followed by the Bonferroni test for parametric variables and expressed as means ± standard deviation. Non-parametric parameters were compared using the Kruskal–Wallis test followed by Dunn’s test and expressed as medians and percentiles. Infarction size was compared by unpaired Student’s *t* test. Mortality was compared using the Goodman test. Statistical analyses were performed using SigmaStat 12.0. The significance level was set at 5%.

## 3. Results

### 3.1. Resistance Exercise Does Not Change Cardiac Remodeling and Ventricular Function in Infarcted Rats

**Figure 1:**
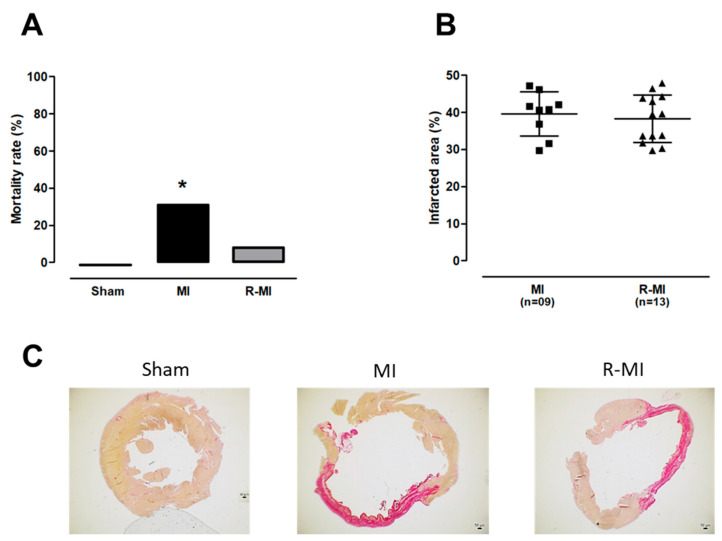
Mortality rate and myocardial infarction size. (**A**) mortality rate from three days after infarction induction surgery until the end of the experiment (data expressed as percentage). (**B**) left ventricle infarcted area size (means, standard deviations and individual values: squares for MI group; triangles for R-MI group). (**C**) representative histological sections of picrosirius red-stained left ventricular myocardium showing infarcted and non-infarcted area. MI: myocardial infarction; R-MI: resistance exercised MI. For A, Goodman test; * *p* < 0.05 vs. Sham; for B, Student’s *t* test; *p* > 0.05.

Mortality rate from three days after infarction induction through to the end of the experiment was significantly higher in MI than Sham; in the R-MI group, mortality rate was between that in MI and Sham and did not differ significantly from either group (Figure 1A). We next evaluated the infarct size, which did not differ between R-MI and MI groups (Figure 1B,C).

**Figure 2:**
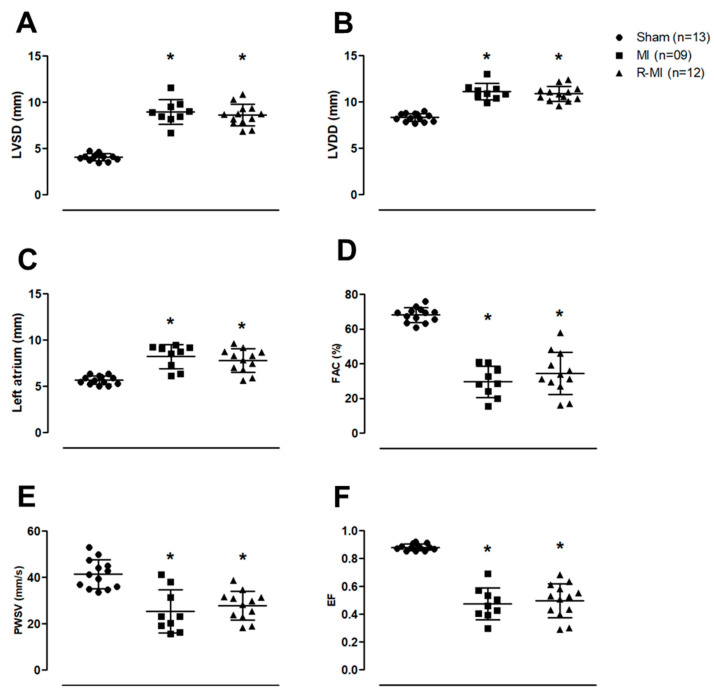
Cardiac structures and left ventricular (LV) function evaluated by echocardiography. (**A**) LV systolic diameter (LVSD); (**B**) LV diastolic diameter (LVDD); (**C**) left atrial diameter; (**D**) fractional area change (FAC); (**E**) LV posterior wall shortening velocity (PWSV); (**F**) ejection fraction (EF); MI: myocardial infarction; R-MI: resistance exercised MI. Data are presented as means, standard deviations, and individual values. ANOVA and Bonferroni; * *p* < 0.05 vs. Sham.

Systemic manifestations induced by cardiac remodeling are only observed when MI size is bigger than 30% of total LV area [55]. Thus, rats that did not reach sizes greater than 30% of the total LV area were excluded from the study. The echocardiogram performed three months after surgery showed that the extent of infarction-induced cardiac injury was similar in R-MI and MI groups (Supplementary data, Table S2). The exercise protocol was initiated after this echocardiogram. Both R-MI and MI groups had LV and left atrial dilation with systolic dysfunction (Figure 2A–F). Training did not change cardiac remodeling, suggesting that resistance exercise can attenuate mortality rate independent of changes in heart structure and function.

### 3.2. Resistance Exercise Preserves Pathways Involved in Skeletal Muscle Protein Turnover

**Figure 3:**
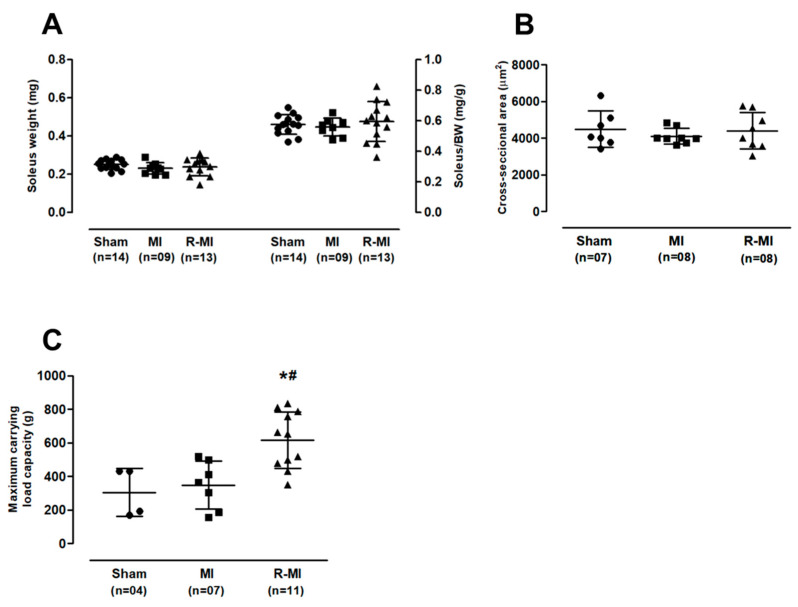
Soleus muscle trophism and maximum carrying load capacity. (**A**) soleus muscle weight; (**B**) soleus cross-sectional area; (**C**) maximum carrying load capacity. BW: body weight; MI: myocardial infarction; R-MI: resistance exercised MI. Data are presented as means, standard deviations, and individual values (circles for Sham group; squares for MI group; triangles for R-MI group). ANOVA and Bonferroni; * *p* < 0.05 vs. Sham; # *p* < 0.05 vs. MI.

Advanced post-infarction-induced cardiac remodeling is often associated with skeletal muscle atrophy [36,38]. Reduced muscle mass is a determinant of decreased exercise capacity and an independent predictor of death in heart failure patients [56,57]. In our study, despite the evident ventricular dysfunction, we did not observe a statistically significant reduction in skeletal muscle mass or the fiber cross-sectional area (Figure 3A,B). It is possible that, despite severely impaired systolic function, heart failure was not clinically prominent in the infarcted rats. Importantly, maximum carrying load capacity was significantly increased in the R-MI group (Figure 3C), showing the improved muscle strength.

**Figure 4:**
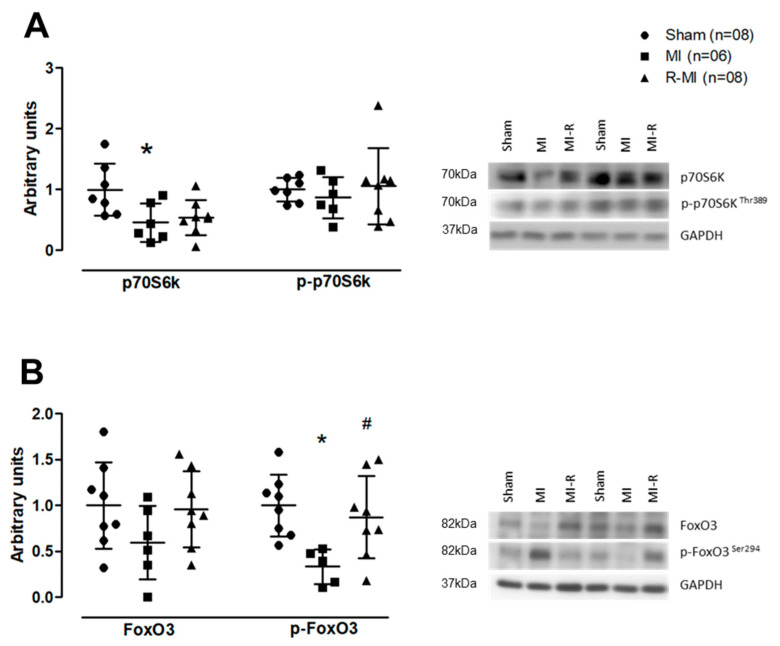
Soleus muscle protein expression. (**A**) total, phosphorylated forms, and representative gels of ribosomal protein S6 kinase beta-1 (p70S6k); (**B**) total, phosphorylated forms, and representative gels of forkhead box O3 (FoxO3). MI: myocardial infarction; R-MI: resistance exercised MI. Graphic data are presented as means, standard deviations, and individual values. ANOVA and Bonferroni; * *p* < 0.05 vs. Sham; # *p* < 0.05 vs. MI.

Skeletal muscle strength can be regulated by various molecular mechanisms, including protein turnover [58]. Thus, we evaluated protein expression of the ribosomal protein S6 kinase beta-1 (S6K1), also known as p70S6 kinase (p70S6k). This protein is involved in protein synthesis and is phosphorylated and activated via mammalian target of rapamycin (mTOR) [59,60]. We observed a 54% reduction in total p70S6k in the MI group; in the R-MI, total p70S6k was between that in Sham and MI groups and did not differ significantly from either group (Figure 4A). No significant difference was observed between groups in p-p70S6k. We next assessed the expression of forkhead box O3, also known as FoxO3, involved in atrophy and protein degradation [61]. Phosphorylated FoxO3 was lower in MI than Sham and R-MI (Figure 4B). Collectively, these results suggest that MI negatively modulated the soleus protein turnover pathway and resistance exercise prevented this change.

### 3.3. Resistance Exercise Modulates Antioxidant Activity and Glycolytic Metabolism in Soleus Muscle of Infarcted Rats

**Figure 5:**
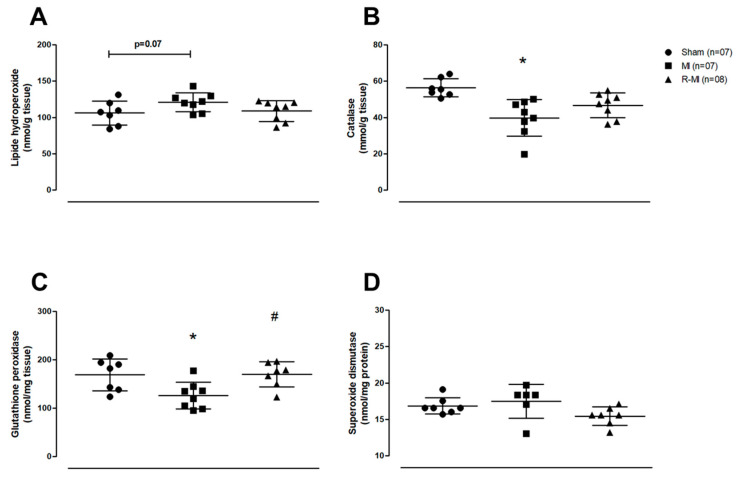
Soleus muscle oxidative stress. (**A**) Lipid hydroperoxide concentration, a marker of oxidative stress; (**B**–**D**) antioxidant enzymes activity. MI: myocardial infarction; R-MI: resistance exercised MI. Data are presented as means, standard deviations, and individual values. ANOVA and Bonferroni; * *p* < 0.05 vs. Sham; # *p* < 0.05 vs. MI.

Myocardial infarction-induced cardiac remodeling is associated with increased reactive oxygen species (ROS) generation and reduced antioxidant enzyme activity in skeletal muscles [37,62]. We observed that soleus lipid hydroperoxide concentration tended to be higher in MI compared to Sham (*p* < 0.07; Figure 5A). Catalase activity was lower in MI than Sham and glutathione peroxidase activity was lower in MI than Sham and R-MI groups (Figure 5B,C).

**Figure 6:**
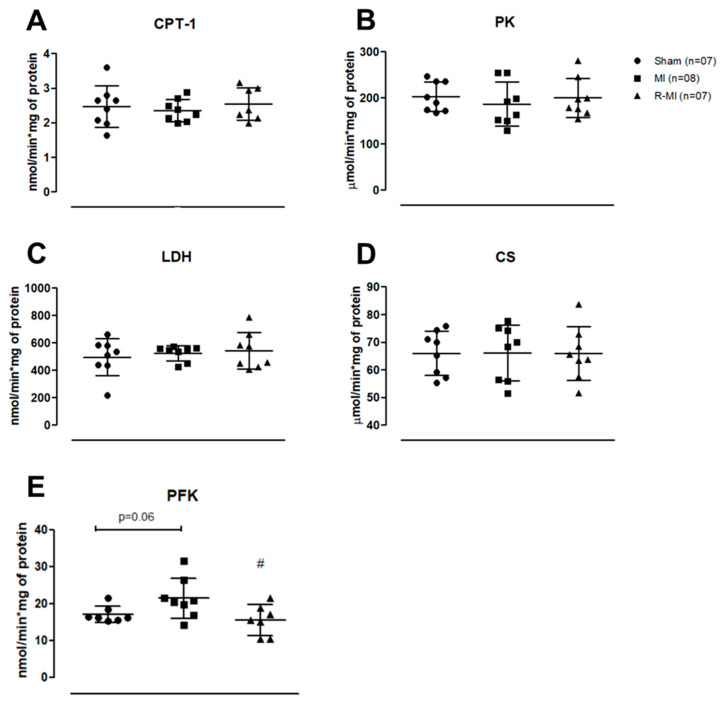
Activity of energy metabolism enzymes in soleus muscle. (**A**) carnitine palmitoyltransferase-1 (CPT-1); (**B**) pyruvato kinase (PK); (**C**) lactate dehydrogenase (LDH); (**D**) citrate synthase (CS); (**E**) phosphofructokinase (PFK). MI: myocardial infarction; R-MI: resistance exercised MI. Data are presented as means, standard deviations, and individual values. ANOVA and Bonferroni; # *p* < 0.05 vs. MI.

In infarcted rats, free fatty acids oxidation is reduced, whereas glycolysis pathway is increased in skeletal muscles [9]. In this study, CPT-1, PK, LDH, and CS activity did not differ between groups (Figure 6A–D). PFK activity was lower in R-MI than MI, and PFK tended (*p* = 0.06) to be higher in MI than Sham (Figure 6E). CPT-1 is responsible for transporting free fatty acids to the mitochondrial membrane [63]. PFK is an important glycolysis pathway enzyme, responsible for converting glucose-6-phosphate into fructose 1,6 bisphosphate from a regulated ATP-dependent process [64]. Together, these data suggest that resistance exercise can preserve antioxidant enzyme activity and reverse the glycolytic phenotype activity observed in infarcted rat soleus muscle.

## 4. Discussion

Physical activity is an important non-pharmacological therapy for treating cardiovascular diseases and their associated complications [22,34]. Impaired skeletal muscle metabolism contributes to increased morbidity during heart failure [28]. Resistance exercise leads to beneficial metabolic effects on the heart and skeletal muscles in healthy subjects [65]. More recently, resistance exercise has been proposed as an adjuvant therapy for cardiovascular disease. However, the mechanisms by which resistance training improves skeletal muscle metabolism and function in different cardiovascular disease models remain unsettled. Here we show that resistance exercise improves soleus energy metabolism and protein turnover, suggesting that resistance exercise can mitigate some of the complications associated with MI.

This work and most studies in rodents have not been designed to evaluate mortality rate induced by MI [36]. As body weight loss usually precedes heart failure development [66], and it was not observed during the experiment, cardiac cachexia or heart failure was not a probable cause of death. It is possible that death was related to cardiac arrythmia. Clinical trials and meta-analyzes have shown that physical training improves exercise tolerance and quality of life and decreases heart failure hospitalizations. However, uncertainty persists about the effects of exercise on mortality [67].

Skeletal muscle weakness and atrophy and reduced aerobic capacity are commonly observed in chronic heart failure [68,69,70]. A growing body of evidence has shown that resistance exercise improves aerobic capacity and skeletal muscle strength in heart failure patients [28,71]. In this study, the fact that 12 weeks of resistance exercise attenuated mortality rate and improved skeletal muscle strength of rats with severe systolic dysfunction shows that this type of exercise is safe after MI and an effective strategy to improve skeletal muscle function.

One mechanism involved in the increased muscle strength induced by resistance exercise is improved neural activation of muscle fibers, which is often accompanied by muscle hypertrophy and enhanced cellular metabolism [72,73,74]. In this study, resistance training improved muscle strength independent of changes in muscle mass or fiber size, suggesting that increased strength may have been driven by adaptations in neural activation and muscle metabolism. We therefore assessed protein expression and phosphorylation of p70S6k and FoxO3, both controlled by the IGF-1/phosphoinositide 3-kinase (PI3K)/protein kinase B (Akt) pathway [59]. This pathway is required for several metabolic processes such as glucose and amino acid transport, and protein synthesis and degradation [75]. PI3K/Akt activation phosphorylates downstream proteins including the mTOR/p70S6k axis to induce protein synthesis and the transcription factor FoxO3, which prevents protein degradation [61,76]. In our study, these pathways were changed in soleus muscle of MI group, as shown by lower p70S6k expression and FoxO3 phosphorylation. Resistance exercise prevented these changes indicating that this exercise modality may rescue protein homeostasis.

Increased oxidative stress is often observed in cardiovascular disease; this is driven by excessive ROS production and/or decreased antioxidant capacity [37,46]. We observed a significant impairment in the activity of two important antioxidant enzymes, catalase and glutathione peroxide, which was prevented by the resistance exercise, suggesting it contributes to muscle health during cardiovascular injury. Changes in energy metabolism after MI have been observed in both cardiac and skeletal muscles [77,78]. Cardiac remodeling after MI is accompanied by a metabolic skeletal myopathy with increased glycolytic enzyme activity [8,9]. PFK is a key enzyme in the regulation of glycolysis, responsible for the irreversible conversion of fructose-6-phosphate and ATP into fructose-1,6-bisphosphate and ADP. In this study, PFK activity was higher in MI than R-MI and Sham (*p* = 0.06 MI vs. Sham), showing that exercise prevented changes in its activity.

The unchanged muscle trophism in our infarcted rats is probably related to the fact that, despite severely impaired LV function, evaluation was performed in an early phase of heart failure. Body weight and skeletal muscle loss are late events in cardiac remodeling and heart failure [68]. We have previously observed that resistance exercise has only improved superoxide dismutase activity and attenuated oxidative stress in the gastrocnemius of infarcted rats. In this study, the same antioxidant potential of resistance exercise was observed in the soleus. Additionally, resistance exercise prevented MI-induced increase in phosphofructokinase activity, a key enzyme in the glycolysis pathway, and changes in proteins involved in protein turnover. Therefore, the main novel data of this study is that the oxidative skeletal musculature is altered molecularly and cellularly by MI and resistance exercise attenuates such changes. Finally, our data allow us to raise the hypothesis that MI alters the protein pathways involved in sustaining protein synthesis and disrupts oxidative metabolism, which may contribute to a later reduction in muscle mass and function; resistance training may prevent metabolic and cell signaling changes, therefore improving quality of life and reducing morbidity after MI.

A limitation of this work is the fact that only male rats were included. Although studies have evaluated the effects of physical exercise in female rats after cardiac injury [79,80], its effects on male vs. female rodents during cardiac remodeling have not been established. Therefore, it is not possible to assume that our results would be valid for females.

## 5. Conclusions

Resistance exercise is safe and increases muscle strength regardless of changes in cardiac structure and function in myocardial-infarcted rats. This exercise modality attenuates soleus glycolytic metabolism changes and improves expression of the proteins required for protein turnover and antioxidant response.

## Data Availability

All data generated or analyzed during this study are included in this Manuscript.

## Supplementary Materials

The following supporting information can be downloaded at: https://www.mdpi.com/article/10.3390/antiox12020291/s1, Table S1: Maximum carrying load capacity evaluated before exercise, after 45 days, and at the end of the experimental period; Table S2. Echocardiographic data before the exercise protocol.
