# Supplementary material for: Effects of Resistance Exercise on Slow-Twitch Soleus Muscle of Infarcted Rats
Source: Antioxidants (Basel). 2023 Jan 27;12(2):291. doi: 10.3390/antiox12020291 (PMC9952394; doi:10.3390/antiox12020291)
Supplement: Supplementary file 1 [file antioxidants-12-00291-s001.zip › antioxidants-2116776-supplementary.pdf]

**Supplementary data**

**Table S1**. Maximum carrying load capacity evaluated before exercise, after 45 days, and at the end of the experimental period

| | Sham | MI | R-MI |
|---|---|---|---|
| **Initial (g)** | 416 ± 90 | 281 ± 70* | 342 ± 86 |
| **45 days (g)** | - | - | 470 ± 148 |
| **Final (g)** | 306 ± 144 | 349 ± 143 | 617 ± 169*# |

MI: myocardial infarction; R-MI: resistance exercised MI. ANOVA and Bonferroni; * p<0.05 vs Sham; # p<0.05 vs MI.

**Table S2.** Echocardiographic data before the exercise protocol

| | Sham | MI | R-MI |
|---|---|---|---|
| **LVSD (mm)** | 4.10 (3.81-4.30) | 8.16 (7.99-8.62) * | 7.89 (7.23-8.55) * |
| **LVDD (mm)** | 7.97 (7.70-8.58) | 10.4 (10.0-10.9) * | 9.79 (9.63-10.4) * |
| **Left atrium diameter (mm)** | 5.29 (5.11-5.66) | 7.66 (6.70-8.27) * | 6.75 (6.27-7.41) * |
| **FAC (%)** | 65.7 ± 5.52 | 40.9 ± 10.3 * | 37.2 ± 9.13 * |
| **PWSV (mm/s)** | 40.5 ± 5.58 | 24.5 ± 7.67 * | 28.6 ± 8.37 * |
| **EF** | 0.86 (0.84-0.89) | 0.51 (0.43-0.57) * | 0.50 (0.42-0.58) * |

MI: myocardial infarction; R-MI: resistance exercised MI; LVSD and LVDD: left ventricle (LV) systolic and diastolic diameters, respectively; FAC: fractional area change; PWSV: LV posterior wall shortening velocity; EF: ejection fraction. ANOVA and Bonferroni or Kruskal-Wallis e Dunn; * p<0.05 vs Sham.
